# Nuclear Receptor Coactivator 2 Promotes Human Breast Cancer Cell Growth by Positively Regulating the MAPK/ERK Pathway

**DOI:** 10.3389/fonc.2019.00164

**Published:** 2019-03-19

**Authors:** Mengjiao Cai, Xin Liang, Xiao Sun, Huan Chen, Yiping Dong, Lingzhi Wu, Suxi Gu, Suxia Han

**Affiliations:** ^1^Department of Oncology, The First Affiliated Hospital of Xi'an Jiaotong University, Xi'an, China; ^2^State Key Laboratory of Proteomics, Beijing Proteome Research Center, Beijing Institute of Radiation Medicine, Beijing, China; ^3^National Center for Protein Sciences, Beijing, China; ^4^Orthopeadic Department, Beijing Tsinghua Changgung Hospital, School of Clinical Medcine, Tsinghua University, Beijing, China

**Keywords:** NCOA2, breast cancer, RASEF, MAPK/ERK, therapeutic target

## Abstract

As a member of the p160 steroid receptor coactivator (SRC) family, nuclear receptor coactivator 2 (NCOA2) is known to play essential roles in many physiological and pathological processes, including development, endocrine regulation, and tumorigenesis. However, the biological function of NCOA2 in breast cancer is not fully understood. We found that the copy number of the *NCOA2* gene was frequently amplified in four breast cancers datasets, varying from 6 to 10%, and the mRNA levels of *NCOA2* were also upregulated in 11% of the sequenced cases/patients (TCGA provisional dataset). Next, we confirmed that *NCOA2* silencing significantly suppressed cell proliferation in different breast cancer cell lines, by inducing cell cycle arrest and apoptosis. Mechanistically, whole-transcriptome sequencing (RNA-Seq) analysis showed that *NCOA2* depletion leads to downregulation of the MAPK/ERK signaling cascade, possibly via downregulating NCOA2's downstream target RASEF. In conclusion, our results suggest NCOA2 as a potential target of therapeutics against breast cancer.

## Introduction

Nuclear receptor coactivator 2 (NCOA2), also known as steroid receptor coactivator 2 (SRC-2), belongs to the p160 steroid receptor coactivator (SRC) family. This protein family contains three members, NCOA1, NCOA2, and NCOA3 (1–3), which are recruited to the enhancer/promoter regions of target genes and serve as transcriptional coactivators for ligand-bound nuclear receptors and transcription factors (4). SRC proteins contain three types of domains: (1) The N-terminal basic helix-loop-helix-Per/ARNT/Sim (bHLH-PAS) domain, which interacts with transcription factors (5, 6); (2) The LXXLL (L for leucine and X for any amino acid) motif, in the central region, responsible for nuclear hormone receptors (NRs) binding (7, 8); (3) Two distinct transcriptional activation domains (AD1 and AD2), at the C-terminus, needed for recruiting additional coregulators, such as histone acetyltransferases (HAT), coactivator-associated arginine methyltransferase 1 (CARM1) and protein arginine methyltransferases (PRMTs) (9–12).

Despite sharing homologous structure with each other, the three SRC members play distinct roles in many physiological and pathological processes, including development, organ function, endocrine regulation, metabolism, and tumorigenesis (13–17). Interestingly, NCOA2 is widely known for its oncogenic role, and *NCOA2* gene fusions, mutations, deletions, and insertions have been observed in multiple cancers including endometrial, cancer, and pleural cancer (18, 19). Additionally, *NCOA2* is amplified or overexpressed in 8% of the primary pancreatic cancers, and its expression level is associated with tumor relapse following androgen deprivation therapy (ADT); mechanistically, NCOA2 overexpression in prostate tumors may lead to hyperactivation of the PI3K/AKT signaling, thus exacerbating tumor malignance (20).

SRC members have also been implicated in the pathology of breast cancer (17) and gene amplification and overexpression of SRCs has been described in breast cancer previously (21–23). NCOA1 has been found to potentiate the roles of estrogen receptor (ER) and mediate transcription reprogramming in ER-positive breast cancer cells (24). However, the biological roles of NCOA2 in breast cancer, especially in triple negative breast cancers (TNBC) remain elusive. In this study, we investigated the role of NCOA2 in regulating cell growth of breast cancer cells with different hormone receptor status.

## Materials and Methods

### Reagents

RPMI-1640 Medium, Dulbecco's modified Eagle's medium (DMEM) and fetal bovine serum (FBS) were obtained from Gibco (Grand Island, NY, USA). Antibodies against phospho-ERK (Thr202/Tyr204; #4370, 1:1,000), ERK (#4695, 1:1,000 WB), phospho-MEK (Ser217/221; #9154, 1:2,000), MEK (#9122, 1:2,000), were purchased from Cell Signaling Technology (Danvers, MA, USA). Antibodies against tubulin (#66240-1-Ig, 1:5,000), β-actin (#60008-1-Ig, 1:5,000), were purchased from Proteintech. The NCOA2 (ab1877, 1:5,000) antibody was purchased from Abcam (Cambridge, UK). Secondary antibodies (1:5,000) conjugated to horseradish peroxidase (HRP) were purchased from Santa Cruz Biotechnology (Santa Cruz, CA, USA).

### Analysis of Publicly Available Datasets

To analyze copy number variation and mRNA expression of the indicated genes in breast cancers, we obtained different dataset by using the cBioPortal (www.cbioportal.org). The copy number of *NCOA2* were analyzed in four independent dataset [METABRIC, Nat Commun 2016; BRCA, INSERM 2016; TCGA Pancancer Atlas; The Metastatic Breast Cancer (MBC) Project]. The mRNA expression of NCOA2 was available in the TCGA provisional dataset. To show the expression levels of NCOA2 in multiple cancer types, we determined the NCOA2 expression in the Ramaswamy Multi-cancer Statistics by using the Oncomine™ online system (www.oncomine.org). To study the effect of *NCOA2* expression on the prognosis of patients with breast cancer, we generated Kaplan-Meier survival curve of breast cancer patients with low or high expression of *NCOA2* by using the Gene Expression Profiling Interactive Analysis (GEPIA) online tool (http://gepia.cancer-pku.cn). GEPIA was also used to analyze the correlation (Pearson correlation coefficient) between *NCOA2* and *RASEF* gene expression.

### Cell Culture, Constructs Preparation, and Transfection

MDA-MB-231, and MCF7 cells were purchased from ATCC. T47D was purchased from Cell Bank of Shanghai Institutes for Biological Sciences of Chinese Academy of Sciences. Cells were cultured in RPMI-1640 Medium or DMEM supplemented with 10% FBS at 37°C in a humidified atmosphere with 5% CO_2_. All constructs used in this study were generated via standard cloning strategy. Briefly, a FLAG-tagged NCOA2 expression vector was created by ligation of NCOA2 open reading frame (ORF) sequence into the lentiviral vector pHAGE (Addgene, MA, USA). Short interfering (si)RNA sequences were as follows: siNCOA2, GGG CTG TTA ACA TTA GC AA; si-negative control (NC), TTC TCC GAA CGT GTC AC GT; siRASEF-1, GCC TTT CTT CAG AGT GAG TTA; siRASEF-2, GCC AAG ATT AAT TCA GCC ATA. For short hairpin (sh)RNA cloning, the oligonucleotides encoding the above indicated siRNA sequences were synthesized with a loop sequence separating the complementary domains, and then cloned into the pLK0.1 vector (Sigma Aldrich, St. Louis, MO, USA). For lentivirus packaging, the recombinant plasmids or control pLKO.1 plasmids were then co-transfected into HEK293T cells with two helper plasmids, psPAX2 and pMD2G (Addgene). Forty-eight hours after transfection, the supernatants containing lentiviruses were harvested and filtered through a 0.45-μm-mesh filter. After lentiviral infection, the infected cells were selected with puromycin (Sigma Aldrich; 4 μg/mL) for 2 days.

### Colony Formation Assays

After lentiviral infection, breast cancer cells were trypsinized and seeded in 6-well plates (1,500 cells/well). After 10 days (when colonies had formed), the plates were rinsed with PBS and the cells were fixed with 4% paraformaldehyde. After 15 min, the cells were stained with crystal violet for 10 min. After washing with PBS for three times, the stained colonies were observed and photographed.

### MTS Assays for Cell Proliferation

After lentiviral infection, breast cancer cells were trypsinized, seeded in 96-well plates (3 × 10^3^ cells/well) and grown at 37°C. At the end of the indicated incubation time, cell proliferation was assessed using an MTS colorimetric assay (Promega, Madison, WI, USA). Upon media removal, MTS (20 μL) was added into the complete culture medium (100 μL) for 30 min. After incubation, the absorbance of the formazan product was recorded at 490 nm using the Spectra MAX 190 microplate reader (Molecular Devices, CA, USA).

### Fluorescence Activated Cell Sorting (FACS)

At the indicated time points, the cells were trypsinized and washed with ice-cold PBS. For cell cycle distribution analysis, the cells were fixed with 70% ethanol and stained with PI/RNase/PBS (100 μg/mL PI and 10 μg/mL RNase A) buffer for 30 min at room temperature (20–25°C) in dark. For apoptosis analysis, the cells were stained using a PI and AnnexinV-FITC staining kit (BD Biosciences) for 30 min at room temperature in the dark. The stained single cell suspension was analyzed on a BD LSRFortessa SORP flow cytometer (BD Biosciences). The ModFit LT software (Verity Software House, Topsham, ME, USA) was used to analyze the flow cytometry data. For apoptotic analysis, the cells in the lower right quadrant are in early apoptosis and those in the upper right quadrant are in mid and late apoptosis. Generally, the two quadrants are scored as total (25).

### Western Blot (WB) Analysis

Total cell lysates were prepared from breast cancer cells using RIPA lysis buffer (Applygen Technologies Inc., Beijing, China) supplemented with a protease inhibitor cocktail (Roche Diagnostics, Mannheim, Germany). Equal amounts of proteins were separated in SDS-polyacrylamide gels and transferred to a polyvinylidene difluoride (PVDF) membrane (Merck Millipore, Massachusetts, USA). The PVDF membrane was incubated with blocking buffer (5% non-fat dry milk in TBST) for 1 h and then with indicated primary antibodies at 4°C overnight. After three washes with TBST, the membrane was incubated with HRP-conjugated secondary antibodies for 1–2 h. Immunoblots were washed and visualized using the SuperSignal™ West Pico Chemiluminescent Substrate (Thermo Fisher Scientific, San Jose, CA, USA) and the ImageQuant LAS500 (GE Healthcare Bio-Sciences AB). The expression of tubulin or β-actin was used as a loading control.

### Reverse Transcription and Quantitative PCR (qPCR)

For reverse transcription, 1 μg total RNA was reverse transcribed using the M-MLV Reverse Transcriptase Kit (Promega) following the manufacturer's protocol. Then following primers were used: Actin-F, 5′- GCA TCC CCC AAA GTT CAC AA-3′, Actin-R, 5′-AGG ACT GGG CCA TTC TCC TT-3′; NCOA2-F, 5′-TGG GGC CTA TGA TGC TTG AG-3′, NCOA2-R, 5′-GGT TTT TGA CAA ATT CCG TGT GG-3′; RASEF-F, 5′-TTC CCC TCA ACC TCT AGG CTA-3′, RASEF-R, 5′-CAA CTT CAC AAT TTG TCC TCT GC-3′. The PCR reactions (20 μL volume) contained: 2 × SYBR® premix Ex Taq™, 10 μL; forward and reverse primers (10 μM), 0.5 μL; cDNA, 4 μL; ddH2O, 5 μL. qPCR was performed on a CFX96 real-time detection system (Bio-Rad) according to the manufacturer's protocol. Each sample was run intriplicate.

### RNA Sequencing and Data Analysis

Whole-transcriptome sequencing (RNA-Seq) was performed as described before (26). Briefly, total RNA was isolated from MDA-MB-231 cells with the QIAGEN RNeasy Plus kit (Qiagen, Valencia, CA, USA). The poly(A)-containing mRNAs were purified and enriched by PCR to create the final library according to the Illumina TruSeq RNA protocols. The libraries were sequenced on an Illumina High HiSeq 2000 with paired-end 100 base pair long reads. The raw sequencing data were examined using the FastQC software (http://www.bioinformatics.babraham.ac.uk/projects/fastqc). The reads were aligned to human genome sequences using TopHat2 (https://ccb.jhu.edu/software/tophat/index.shtml). Mapped reads were assembled with the Cufflinks (http://cole-trapnell-lab.github.io/cufflinks/). Read count per RNA was computed using HTSeq (https://htseq.readthedocs.io/en/release_0.10.0/). Log2 transformations were performed on normalized read counts. Differential expression analyses were performed using DESeq2 (https://bioconductor.riken.jp/packages/2.14/bioc/html/DESeq2.html). For functional annotation, the KEGG pathways were determined by analyzing dysregulated mRNAs using DAVID (https://david.ncifcrf.gov). Gene Set Enrichment Analysis (GSEA) was performed to evaluate significant enrichment of genes using GSEA software (http://software.broadinstitute.org/gsea/index.jsp).

### Statistical Analysis

For statistical analysis, the Student's *t*-test was used for parametric variables. All experiments were performed at least three times, and a *P* < 0.05 was considered statistically significant.

## Results

### Analysis of the *NCOA2* Gene in Breast Cancer Samples

**Figure 1:**
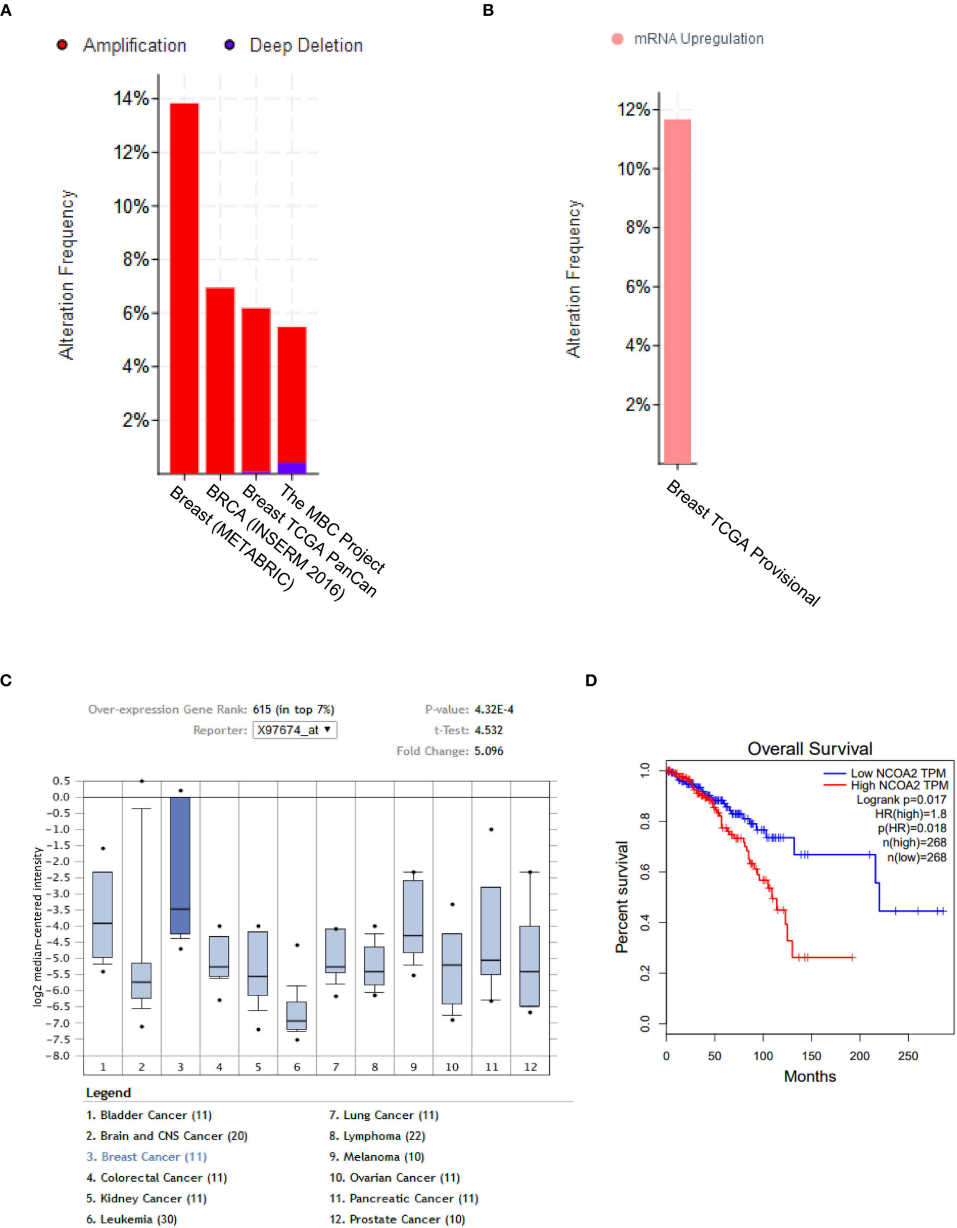
NCOA2 is amplified in breast cancer. **(A)** The copy number of *NCOA2* were analyzed in four independent dataset [METABRIC, Nat Commun 2016; BRCA, INSERM 2016; TCGA Pancancer Atlas; The Metastatic Breast Cancer (MBC) Project] using the cBioPortal online tool. The amplification frequency in each dataset was shown in red bar. **(B)** The mRNA expression of NCOA2 was available in the TCGA provisional dataset using the cBioPortal online tool. The frequency of NCOA2 high expression was shown in pink. **(C)** The mRNA expression levels of *NCOA2* in multiple cancer types (in Ramaswamy Multi-cancer Statistics) were determined by using the Oncomine online dataset. In the single-gene views of the online system, samples are divided between the breast cancer (test) class and the other cancer type (control) classes against which the NCOA2 gene expression in the test class is measured. The breast cancer (test) class is highlighted in deeper blue and statistics including fold change and *p*-value were presented in the top right corner. **(D)** Overall survival (OS) rates were compared among patients in different quartiles using Kaplan–Meier survival curves and log-rank tests by using the GEPIA online tool.

The copy number of the *NCOA2* gene was analyzed by using the cBioPortal online tool (www.cbioportal.org) and found to be frequently amplified in four independent breast cancers datasets [METABRIC, Nat Commun 2016; BRCA, INSERM 2016; TCGA Pancancer Atlas; The Metastatic Breast Cancer (MBC) Project] (27–29), varying from 5 to 14% amplification (Figure 1A). Not surprisingly, the mRNA levels of *NCOA2* were also upregulated in 118 (11%) of 1,082 sequenced cases/patients (TCGA provisional dataset; Figure 1B). Moreover, by cross-comparing the results obtained from the Ramaswamy Multi-cancer Statistics using the online Oncomine tool (www.oncomine.org), we found that the expression of *NCOA2* in breast cancer was relatively higher than that in other cancer types, including prostate cancer, bladder cancer, lung cancer, and lymphoma among others (Figure 1C) (30), suggesting an important role for *NCOA2* in regulating breast cancer development. Importantly, as revealed in Figure 1D, higher *NCOA2* expression was significantly correlated with poor overall survival of patients with breast cancer, with a hazard ratio of 1.8 (log-rank *p* = 0.017).

### NCOA2 Is Essential for the Growth of Breast Cancer Cells

**Figure 2:**
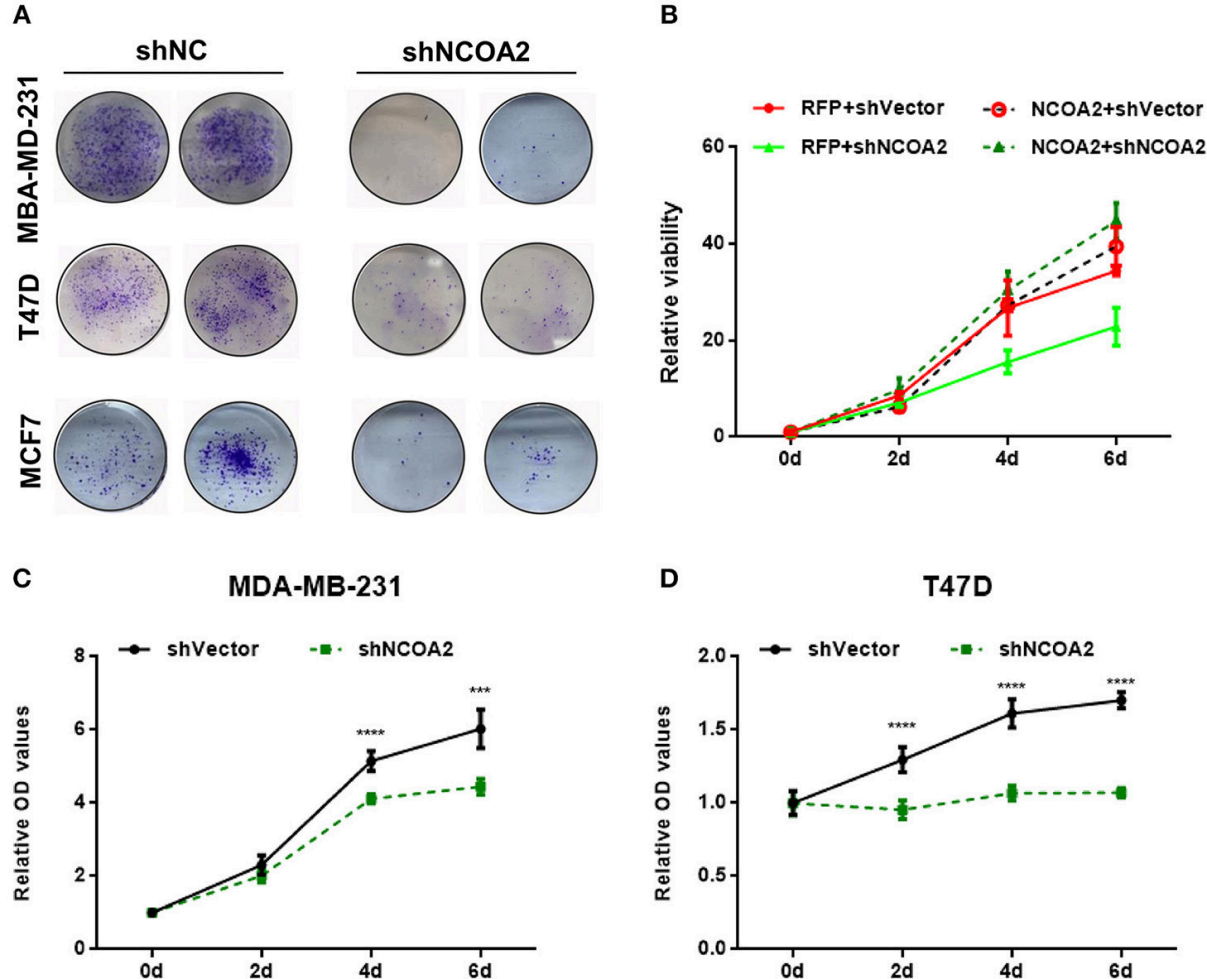
Knockdown of *NCOA2* significantly inhibits breast cancer cell growth. **(A)** MDA-MB-231, T47D, and MCF7 cells overexpressing the indicated shRNAs were grown for 10 days, to allow the formation of colonies, which were then stained and photographed. **(B)** *NCOA2* silencing-resistant MDA-MB-231 cells (dashed lines, referred to as NCOA2) or RFP-overexpressing cells (control group; continuous lines, referred to as RFP) were infected with viruses overexpressing shNCOA2 or control shRNAs, and the cell growth was assessed in MTS proliferation assays. **(C,D)** MTS cell proliferation assays were performed on MDA-MB-231 **(C)** and T47D cells **(D)**, after overexpression of shNCOA2 or control shRNAs. ***P < 0.001; ****P < 0.0001.

To evaluate the biological role of NCOA2 in human breast cancer, we applied lentivirus-mediated RNA interference toward *NCOA2* in three breast cancer cell lines (MDA-MB-231, ERα-, PR–, and HER2–; T47D, ERα+, PR+, and HER2–; MCF7, ERα+, PR+, and HER2+/–). Both colony formation (Figure 2A) and MTS cell proliferation (Figures 2C,D) assays indicated that *NCOA2* knockdown significantly suppressed cell proliferation in different breast cancer cell lines. To rule out the unwanted toxicity induced by the off-target activity of the interfering RNAs (31) we generated a NCOA2-overexpressing construct with a silent mutation that made *NCOA2* mRNA resistant to RNA interference. Ectopic expression of shRNA-resistant NCOA2 (dashed line, green) completely rescued the inhibitory effect of shNCOA2 treatment in MDA-MB-231 cells, demonstrating that the inhibition of cell growth resulted from *NCOA2* depletion (Figure 2B).

**Figure 3:**
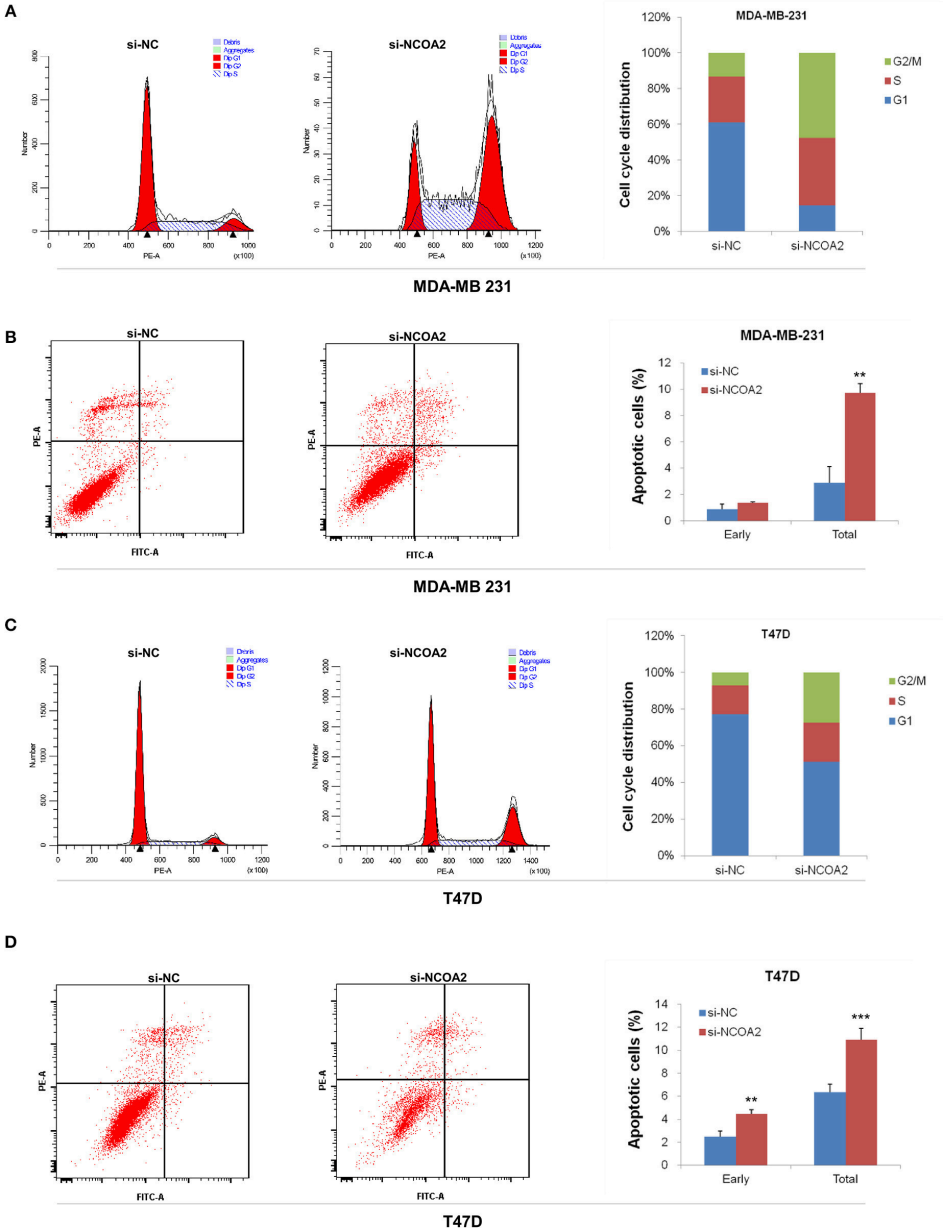
Knockdown of *NCOA2* inhibits cell cycle and induces apoptosis. **(A,B)** MDA-MB-231 cells were subjected to PI **(A)** or AnnexinV-FITC **(B)** staining, for cell cycle or apoptosis detection, respectively. **(C,D)** T47D cells were subjected to PI **(C)** or AnnexinV-FITC **(D)** staining, for cell cycle or apoptosis detection, respectively. For apoptotic analysis, the cells in the lower right quadrant are in early apoptosis and those in the upper right quadrant are in mid and late apoptosis. Generally, the two quadrants are scored as total. **P < 0.01; ***P < 0.001.

Both *NCOA2*-depleted MDA-MB-231 and T47D cells showed a G2/M arrest, as demonstrated by cell cycle analysis (Figures 3A,C). At the same time, we detected significant apoptosis in these cells (Figures 3B,D). These results indicate that *NCOA2* knockdown hampers breast cancer cells proliferation primarily by inducing cell cycle arrest and apoptosis.

### NCOA2 Regulates the Mitogen-Activated Protein Kinase MAPK/ERK Signaling Pathway

**Figure 4:**
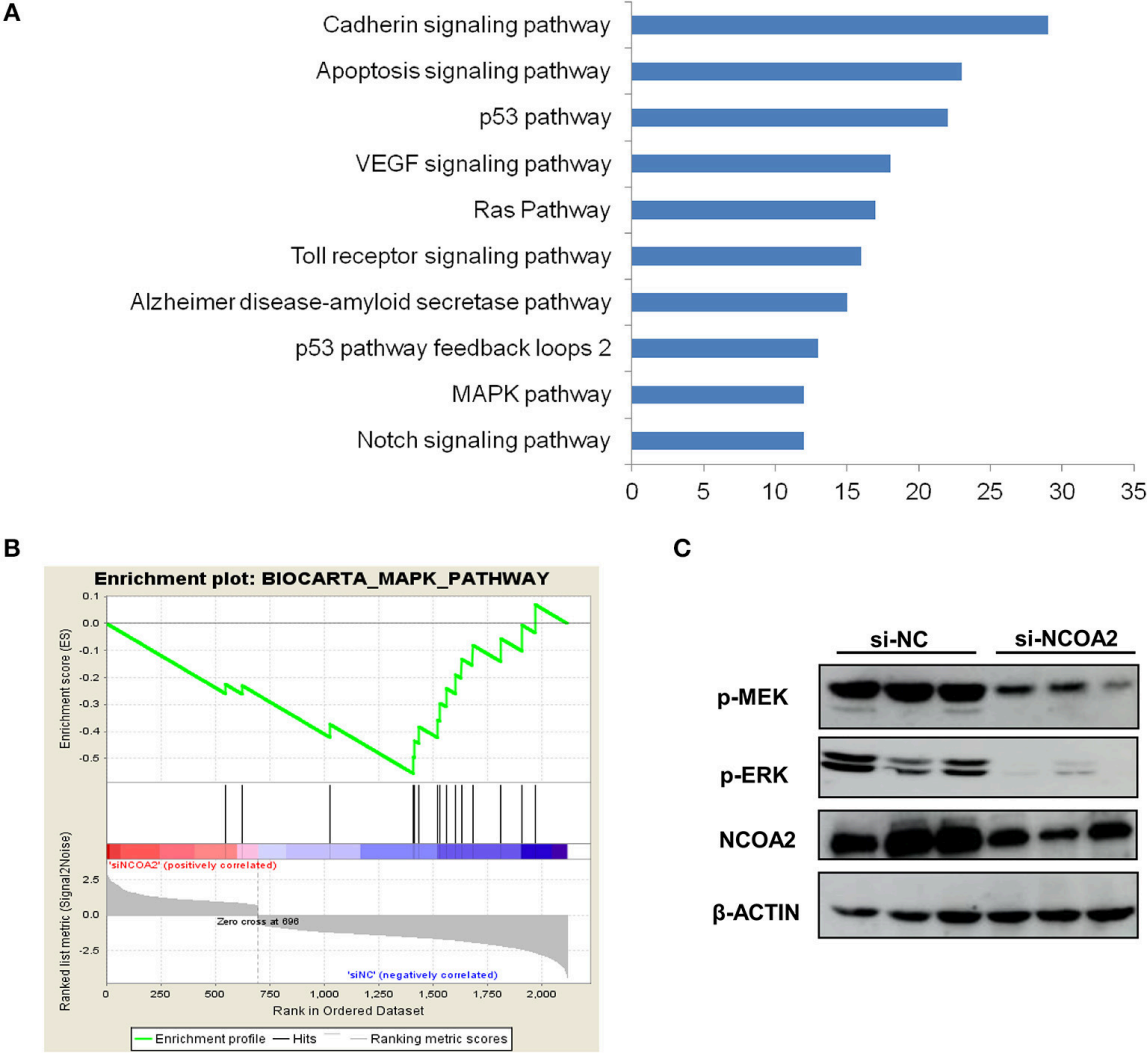
*NCOA2* depletion suppresses the MAPK/ERK signaling cascade. **(A)** RNA-Seq was performed on *NCOA2*-depleted MDA-MB-231 cells; differentially expressed genes were subjected to pathway analysis using the DAVID online tool. The top 10 significant pathways are shown. **(B)** GSEA analysis was performed to evaluate significant enrichment of genes on the MAPK pathway. **(C)** WB analysis of MDA-MB-231 cells after *NCOA2* knockdown to assess the expression of pMEK and pERK.

To characterize the functional effects of *NCOA2* knockdown, we assessed the gene expression profiles of *NCOA2*-depleted MDA-MB-231 cells by RNA-Seq. We identified 696 upregulated and 1,421 downregulated genes in *NCOA2*-depleted cells, as compared with the control group. All differentially expressed genes were subjected to pathway analysis using the DAVID online tool. Our data indicated that NCOA2 is implicated in the regulation of several essential pathways, including the apoptotic, p53, MAPK, and Ras pathways among others (Figure 4A). Next, GSEA analysis revealed that genes dysregulated upon *NCOA2* depletion were enriched in the MAPK signaling pathway (Figure 4B). Furthermore, WB analysis confirmed that when *NCOA2* was depleted in breast cancer cells, the activity of the MAPK/ERK signaling was strongly inhibited, as represented by the decreased protein levels of p-MEK and p-ERK (Figure 4C).

### NCOA2-Controlled *RASEF* Expression May Regulate ERK Activation and Cell Proliferation

**Figure 5:**
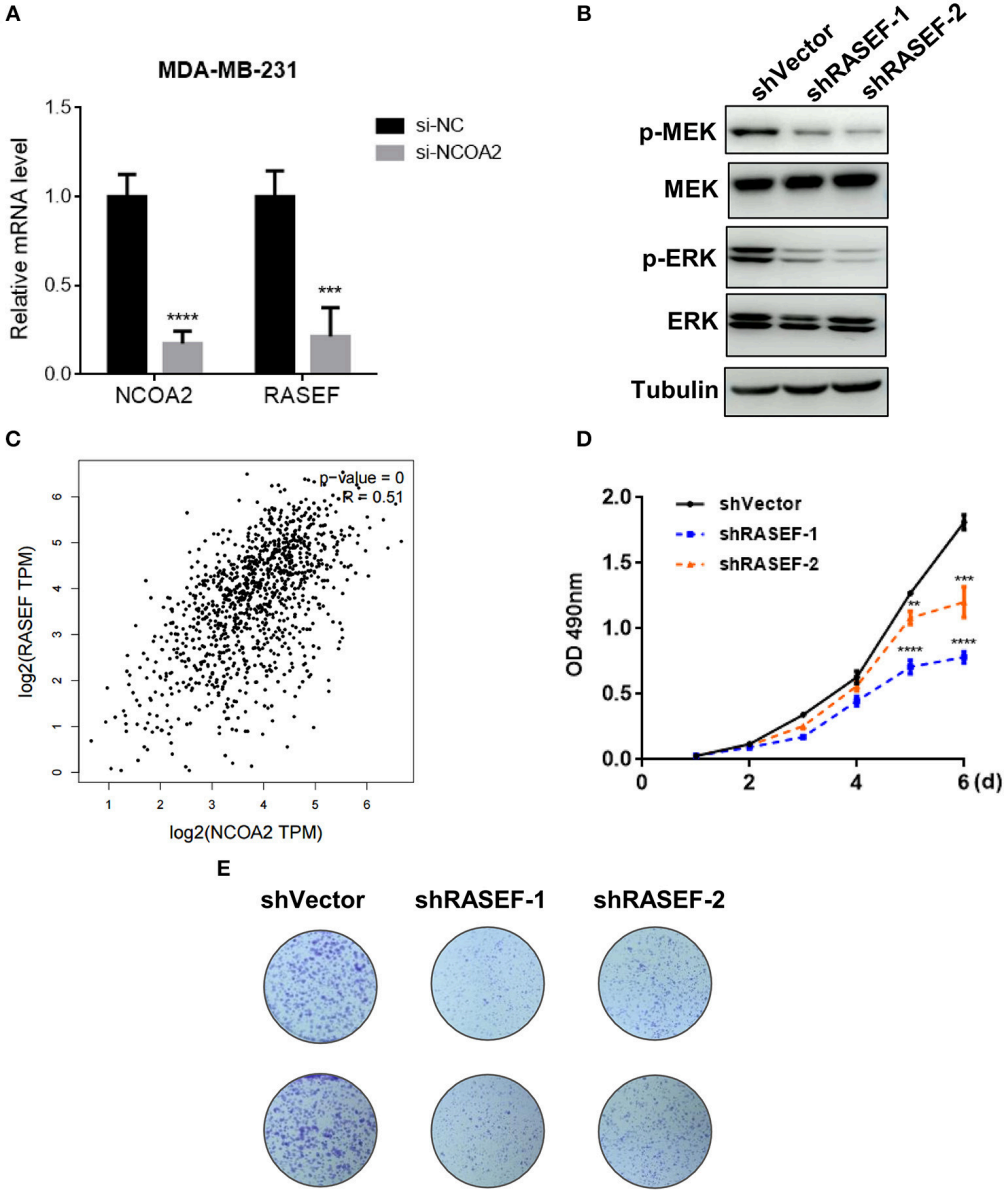
*NCOA2* depletion inactivates the MAPK/ERK signaling cascade by downregulating RASEF expression. **(A)** The mRNA expression levels of *NCOA2* and *RASEF* were determined in *NCOA2*-depleted and control MDA-MB-231 cells. **(B)** WB analysis of *RASEF*-silenced MDA-MB-231 cells to assess the expression of pMEK and pERK. **(C)** The correlation (Pearson correlation coefficient) between *NCOA2* and *RASEF* expression in breast cancer specimens was assessed using the GEPIA online tool on the TCGA database. **(D,E)** MTS cell proliferation **(D)** and colony formation **(E)** assays were performed on MDA-MB-231 cells after overexpression of shRASEF or control shRNAs. ***P < 0.001; ****P < 0.0001.

The Ras superfamily of small GTPases consists of more than 100 members, which can be divided into several principal families: Ras, Rho, Rab, Arf (ADP-ribosylation factors), and Ran (32). Studies have shown that many Ras superfamily members are involved in the regulation of the MAPK/ERK signaling pathway, which is an oncogenic signaling cascade essential for cancer cell growth (33–35). Interestingly, we identified that the expression of *Ras* and EF-hand domain containing (*RASEF*) was significantly downregulated upon *NCOA2* depletion, as shown by RNA sequencing (Figure 4A) and qPCR analysis (Figure 5A). RASEF, a member of the Rab GTPase protein family, has been shown to positively regulate ERK signaling cascade in lung cancer (36). We confirmed that *RASEF* depletion could remarkably suppress the activity of the MAPK/ERK signaling cascade by using two independent shRNA sequences targeting *RASEF* (Figure 5B). Further, analysis of breast cancer tissue data from the TCGA database showed that *RASEF* and *NCOA2* levels were strongly and positively correlated (*R* = 0.51, *P* < 0.0001; Figure 5C). Further, to determine whether *RASEF* downregulation accounted for the anti-proliferative effect of *NCOA2* depletion, we assessed breast cancer cell growth after *RASEF* knockdown in MTS cell proliferation (Figure 5D) and colony formation (Figure 5E) assays. We found that *RASEF* depletion significantly inhibited the growth of MDA-MB-231 cells. Therefore, our data suggested that *RASEF* expression is controlled by NCOA2, and decreased RASEF levels might contribute to ERK deactivation and to the blockade of cell growth.

## Discussion

Breast cancer is a common malignant cancer occurring in the breast epithelial tissue and remains one of the diseases with the highest mortality rate. Statistically, one in eight women in the US may develop breast cancer in her lifetime, and over 30,000 breast cancer deaths were expected to occur among US women in 2013 (37). Numerous studies conducted on breast cancer have revealed that breast cancer is a complex heterogeneous disease, which can be divided into a variety of subtypes with different responses to the treatment and clinical outcomes (38). At the molecular level, differences in the expression levels of the established breast cancer biomarkers ER, PR, and HER2 are used for decision-making in clinical investigations (39, 40). ER and PR are well-known nuclear receptors that can interact with nuclear receptor co-activators, including SRC family members, to initiate transcription of steroid-responsive genes (41). Given their importance in NR signaling, the SRC family (NCOA1-3) is worthy of extensive investigation.

While the functions of NCOA1 and NCOA3 have been widely explored in breast cancer, little is known about the biological roles of NCOA2 in regulating genes involved in breast cancer progression (23, 42–44). Although *NCOA2* depletion in MCF-7 cells has been shown to suppress estrogen-dependent ERα transactivation function, it does not seem to affect the estrogen-dependent proliferation of these cells (45), suggesting an ERα-independent role for NCOA2 may exist in breast cancer.

Here, we showed that *NCOA2* is amplified and overexpressed in around 10% breast cancer samples in the TCGA data set, and higher *NCOA2* expression correlated with a poor overall survival status (Figure 1). In *in vitro* experiments in breast cancer cell lines with different nuclear receptor status (MDA-MB-231, ERα-, PR–, and HER2–; T47D, ERα+, PR+, and HER2-; MCF7, ERα+, PR+, and HER2+/–), we demonstrated that *NCOA2* knockdown strongly inhibited cancer cell growth. Previous studies have established that the differential expression of ER and SRC proteins modulates estrogenic action during tumorigenesis of breast cancer: in a group of breast cancer specimens, the expression of ERα significantly correlated with that of PR and nuclear receptor corepressor 1 (NCoR1), whereas ERβ expression was associated with NCOA1 and NCOA2 expression (46). On the other hand, increased levels of NCOA3 have been shown to favor the functional interaction of ERα and promote the estrogen-dependent mitogenic stimulation of breast cancer cells (47). Since the MDA-MB-231 line is an ERβ-positive cell line, it is worth further investigation to explorer the interaction between NCOA2 and ERβ receptor. Additional functional and biochemical studies are necessary to clarify how NCOA2 interacts with different NRs, such as ERβ, androgen receptors, and corticosteroid receptors.

Mechanistically, we performed RNA-Seq analysis to explore the potential mechanisms regulated by NCOA2 in MDA-MB-231 cells. Our data show that NCOA2 may regulate TNBC cell growth by modulating the oncogenic MAPK/ERK signaling pathway (Figure 4), possibly via the downregulation of its downstream target RASEF (Figure 5). RASEF has been reported to be a novel diagnostic biomarker for lung cancer and play an oncogenic role in lung cancer cell growth, possibly by activating the ERK signaling cascade (36).

In summary, our findings show that NCOA2 is frequently amplified in breast cancer and loss of NCOA2 remarkably attenuates cell growth in breast cancer cell lines with different NR status, strongly indicating that NCOA2 might be a potential target for breast cancer treatment. The comprehensive molecular mechanism underlying the interaction between NCOA2 and different NRs is worthy of systemic investigation.

## Data Availability

All datasets generated for this study are included in the manuscript and/or the supplementary files.

## Author Contributions

SH and SG conceived and designed the study. MC, XL, and XS performed the experiments. MC, HC, and SG analyzed the data. YD and LW contributed reagents, materials, and analysis tools. HC wrote the manuscript.

### Conflict of Interest Statement

The authors declare that the research was conducted in the absence of any commercial or financial relationships that could be construed as a potential conflict of interest.
